# Network meta-analysis and dose–response analysis of exercise on sleep quality and BMI in obese populations

**DOI:** 10.3389/fpubh.2026.1766438

**Published:** 2026-02-12

**Authors:** Hongfei Wang, Jiaxin Wang, Lunan Zhao, Yongliang Zhu

**Affiliations:** 1 College of Physical Education and Sport Science, Qufu Normal University, Qufu, China; 2 School of Competitive Sport, Shandong Sport University, Rizhao, China

**Keywords:** body mass index, dose–response relationship, exercise, network meta-analysis, obesity, randomized controlled trials, sleep quality

## Abstract

**Background and aims:**

Exercise is key to ameliorating sleep disorders in obese populations; however, the relative benefits of different exercise modes and the optimal dosage remain unclear. This study aims to systematically evaluate the relative efficacy and dose–response characteristics of seven exercise interventions on sleep quality (Pittsburgh Sleep Quality Index [PSQI]) and body mass index (BMI) in overweight and obese populations via network meta-analysis (NMA).

**Methods:**

This study strictly adhered to the Preferred Reporting Items for Systematic Reviews and Meta-Analyses (PRISMA) guidelines and systematically searched nine databases, including PubMed, Embase, and China National Knowledge Infrastructure (CNKI), from inception to 15 October 2025. Randomized controlled trials (RCTs) evaluating the effects of the aforementioned exercise interventions on sleep quality (primarily PSQI) or body mass index (BMI) in overweight or obese individuals were included. Network meta-analysis was conducted using Stata 18.0 software. The standardized mean difference (SMD) was used as the effect size, and interventions were ranked using the Surface Under the Cumulative RAnking curve (SUCRA).

**Results:**

The network meta-analysis revealed that combined aerobic and resistance training (ART, SUCRA ≈ 77.1%) and resistance training (RT, SUCRA ≈ 75.2%) were significantly superior to other interventions in improving sleep quality. Conversely, erobic Exercise (AE, SUCRA ≈ 74.0%) was most effective in reducing BMI. Dose-effect analysis indicated that improvements in sleep quality did not exhibit significant linear dose-dependency (*p* > 0.05). However, BMI improvement showed a significant non-linear “U-shaped” dose–response relationship (p_quadratic = 0.009). The fitted curve suggests that a cumulative intervention duration of 60–70 h represents the optimal dosage window for weight loss, with diminishing marginal returns observed beyond this range.

**Conclusion:**

Exercise interventions demonstrate significant specificity in their benefits for health outcomes in obese populations. Resistance and combined training are recommended as the preferred strategies for improving sleep, with benefits depending more on the mode than on high cumulative dosage. Conversely, aerobic exercise is the optimal protocol for weight loss, provided the exercise volume is controlled within the optimal dosage window to maximize returns. These findings offer evidence-based grounds for clinical practice to formulate differentiated and precise exercise prescriptions for obesity and sleep management.

**Systematic review registration:**

www.crd.york.ac.uk/prospero, identifier CRD420251251401.

## 1 Introduction

Obesity has evolved into a global public health crisis. A 2017 World Health Organization report stated that over 4 million people die annually as a result of being overweight or obese (1). It is projected that by 2030, one billion people will suffer from obesity (2). Obesity is not only an independent risk factor for chronic conditions, such as type 2 diabetes and cardiovascular disease, but also exists in a vicious cycle of mutual causality with sleep disorders (3–5). Epidemiological evidence indicates that the prevalence of poor subjective sleep quality, short sleep duration, and obstructive sleep apnea (OSA) is significantly higher in obese populations compared to those with normal weight (6). Notably, while OSA is a common pathological change in obese populations, clinical patient complaints often focus more on subjective manifestations of declined sleep quality, such as difficulty initiating sleep, sleep maintenance disorders, and impaired daytime function. Research shows that the objective Apnea-Hypopnea Index (AHI) does not always parallel the patient’s subjective sleep perception (e.g., Pittsburgh Sleep Quality Index [PSQI] scores) (7–9), implying that focusing solely on pathological indices may not fully reflect the patient’s sleep quality.

Non-pharmacological interventions, particularly exercise interventions, have been shown to be effective in improving sleep quality in obese populations (10). For instance, a systematic review and meta-analysis by Lin et al. (9) on patients with OSA found that in the obese subgroup (body mass index [BMI] ≥ 30 kg/m^2^), exercise intervention significantly reduced the AHI and Epworth Sleepiness Scale (ESS) scores, while also improving BMI. This confirms that exercise can effectively ameliorate sleep-disordered breathing in obese populations. Similarly, a systematic review and meta-analysis by Peng et al. (11) on OSA patients found that exercise intervention significantly reduced AHI and effectively improved ESS and sleep quality (PSQI); however, there was no significant difference in BMI between the intervention and control groups.

Existing meta-analyses mostly focus on comparing single exercise modes with blank controls or performing simple pairwise comparisons. The relative effectiveness of different exercise modes remains unclear. Currently, there is a lack of systematic full-network comparisons within the same framework that include mainstream conventional exercises, mind–body exercises with Eastern characteristics and core stability training. This leaves clinicians unable to judge, based on evidence, which exercise mode offers optimal benefits for improving sleep in obese populations when faced with multiple options. Secondly, the dose–response relationship of exercise interventions remains unclear. Clinically, it is not yet known whether an optimal exercise frequency or duration threshold exists to maximize improvements in sleep quality for obese patients.

In view of this, the present study intends to employ network meta-analysis (NMA) to systematically evaluate the relative effects of seven exercise interventions—combined aerobic and resistance training (ART), resistance training (RT), physical activity (PA), Baduanjin, aerobic exercise (AE), Pilates, and yoga—on subjective sleep quality (PSQI) and body mass index (BMI) in overweight and obese populations within the same comparative system. This study aims to reveal the hierarchy of advantages and disadvantages of different exercise modes and their optimal dosage characteristics, explaining how exercise benefits patients through the dual pathways of improving subjective perception and body composition, thereby providing high-quality evidence for the clinical formulation of personalized and precise exercise prescriptions for obesity and sleep management.

## 2 Materials and methods

This study protocol has been registered at PROSPERO (Registration Number: CRD420251251401), specifying the research objectives, inclusion and exclusion criteria, interventions, control measures, and planned outcome assessments. The implementation of this systematic review strictly followed the pre-registered protocol and adhered to the Preferred Reporting Items for Systematic Reviews and Meta-Analyses (PRISMA) checklist for implementation and reporting (12).

### 2.1 Search strategy

We systematically searched PubMed, Embase, Cumulative Index to Nursing and Allied Health Literature (CINAHL), Web of Science, Scopus, Cochrane Library, China National Knowledge Infrastructure (CNKI), Wanfang, and Virtual IP (VIP) databases from inception to 15 October 2025. The search strategy combined Medical Subject Headings (MeSH) terms and free text words, developed based on PubMed and adapted for other databases. Additionally, we manually searched the reference lists of all included studies to ensure maximum retrieval of relevant research. Details are listed in Table 1.

**Table 1:** Database search strategy.

Search combination	Search term	Search field
#1	Obesity OR overweight	MeSH terms
#2	Obese OR obesity OR overweight OR adiposity OR body mass index OR BMI	Title/abstract
#3	Exercise OR physical activity OR resistance training OR yoga OR Pilates OR mind–body therapies	MeSH terms
#4	exercise OR “physical activity” OR training OR aerobic OR resistance OR strength OR combined OR concurrent OR yoga OR Pilates OR Baduanjin OR “Tai Chi” OR aerobics	Title/abstract
#5	Randomized controlled trial OR controlled clinical trial	Publication type
#6	randomized OR randomized OR RCT OR placebo OR controlled trial OR clinical trial	Title/abstract
#7	(#1 OR #2) AND (#3 OR #4) AND (#5 OR #6)	

### 2.2 Inclusion and exclusion criteria

Two reviewers independently screened the titles and abstracts of the literature, followed by a full-text review of potentially eligible articles to determine final inclusion. Any disagreements were resolved through discussion or third-party arbitration. Inclusion criteria were established based on the Population, Intervention, Comparator, Outcome, Study Design (PICO-S) framework: (1) Population (P): Individuals diagnosed as overweight or obese; (2) Intervention (I): Any form of exercise intervention, including but not limited to Combined Training (ART), Resistance Training (RT), physical activity (PA), Baduanjin, aerobic exercise (AE), Pilates, and yoga; (3) Comparator (C): Blank control or sham intervention; studies combining diet interventions where dietary protocols differed between groups were excluded; (4) Outcome (O): Reporting at least one of the following indicators: sleep quality (primarily Pittsburgh Sleep Quality Index, PSQI) or Body Mass Index (BMI); (5) Study Design (S): randomized controlled trials (RCTs). Exclusion criteria included: non-randomized controlled trials, duplicate publications, mismatched outcome indicators, mismatched interventions, incomplete data descriptions, and mismatched control group settings (e.g., non-traditional control groups).

### 2.3 Data collection

Two researchers independently performed literature screening and data extraction. First, titles and abstracts were read for initial screening, followed by full-text retrieval for secondary screening to determine final inclusion. Any disagreements were resolved through discussion or consultation with a third party. A pre-designed data extraction form was used to extract the following information: (1) Basic Information: First author, year of publication. (2) Population Characteristics: Sample size, age, baseline BMI. (3) Intervention Characteristics: Type of exercise intervention, control group measures, intervention frequency, training intensity, single session duration, total intervention period, and total exercise dosage. (4) Outcome Indicators: Means and standard deviations for PSQI and BMI. For studies with incomplete data, attempts were made to contact the original authors; if unavailable, data were converted or estimated according to methods recommended by the *Cochrane Handbook*.

### 2.4 Risk of bias and certainty of evidence

We used the Cochrane Collaboration’s Risk of Bias 2.0 (RoB 2) tool to evaluate the quality of included studies (13). The assessment covered five domains: bias arising from the randomization process (D1), bias due to deviations from intended interventions (D2), bias due to missing outcome data (D3), bias in measurement of the outcome (D4), and bias in selection of the reported result (D5). Each domain was judged as “Low Risk,” “Some Concerns,” or “High Risk,” yielding an overall risk of bias judgment for each study. The Grading of Recommendations Assessment, Development, and Evaluation (GRADE) system was employed to evaluate the quality of evidence (14, 15). Evidence was downgraded based on five aspects: risk of bias in study design, inconsistency, indirectness, imprecision, and publication bias. The quality of evidence was classified into four levels: High, Moderate, Low, and Very Low. Two reviewers independently completed the quality assessment, reaching consensus through discussion where opinions differed, or consulting third-party expert opinions when necessary.

### 2.5 Data analysis

This study employed network meta-analysis (NMA). Data synthesis was performed using the network meta package in Stata 18.0 software. As the outcome indicators (PSQI, BMI) were continuous variables, the standardized mean difference (SMD) and its 95% confidence interval (CI) were used as the effect size, with the significance level set at *α* = 0.05. First, a network plot was constructed to display direct comparison relationships between interventions. For closed-loop network structures, node analysis was used for global inconsistency testing; if *p* > 0.05, consistency was considered good, and a consistency model was used for calculation. Simultaneously, the Node-splitting method was used to assess local inconsistency; if *p* < 0.05, it suggested significant inconsistency. The Surface Under the Cumulative RAnking curve (SUCRA) was used to rank the relative efficacy of all interventions. SUCRA values range from 0 to 100%, with values closer to 100% indicating a higher probability that the intervention is the best measure. Adjusted comparison funnel plots and Egger’s regression were used to test for potential publication bias. Dose Calculation Method: To quantify the total load of the exercise intervention, this study calculated the cumulative exercise duration over the entire intervention period. The calculation formula is as follows: Total Dosage = W × F × 60/T, where W represents the intervention duration in weeks, F represents the weekly training frequency, and T represents the duration of a single session (16). For studies reporting a frequency range (e.g., 5–6 times/week), the mean value of that range was used for calculation. For interventions including warm-up and cool-down activities, if the literature explicitly reported the total session duration, it was counted in full toward the total dosage. This calculation result was used for subsequent dose–response meta-regression analysis.

## 3 Results

### 3.1 Literature identification and selection process

**Figure 1:**
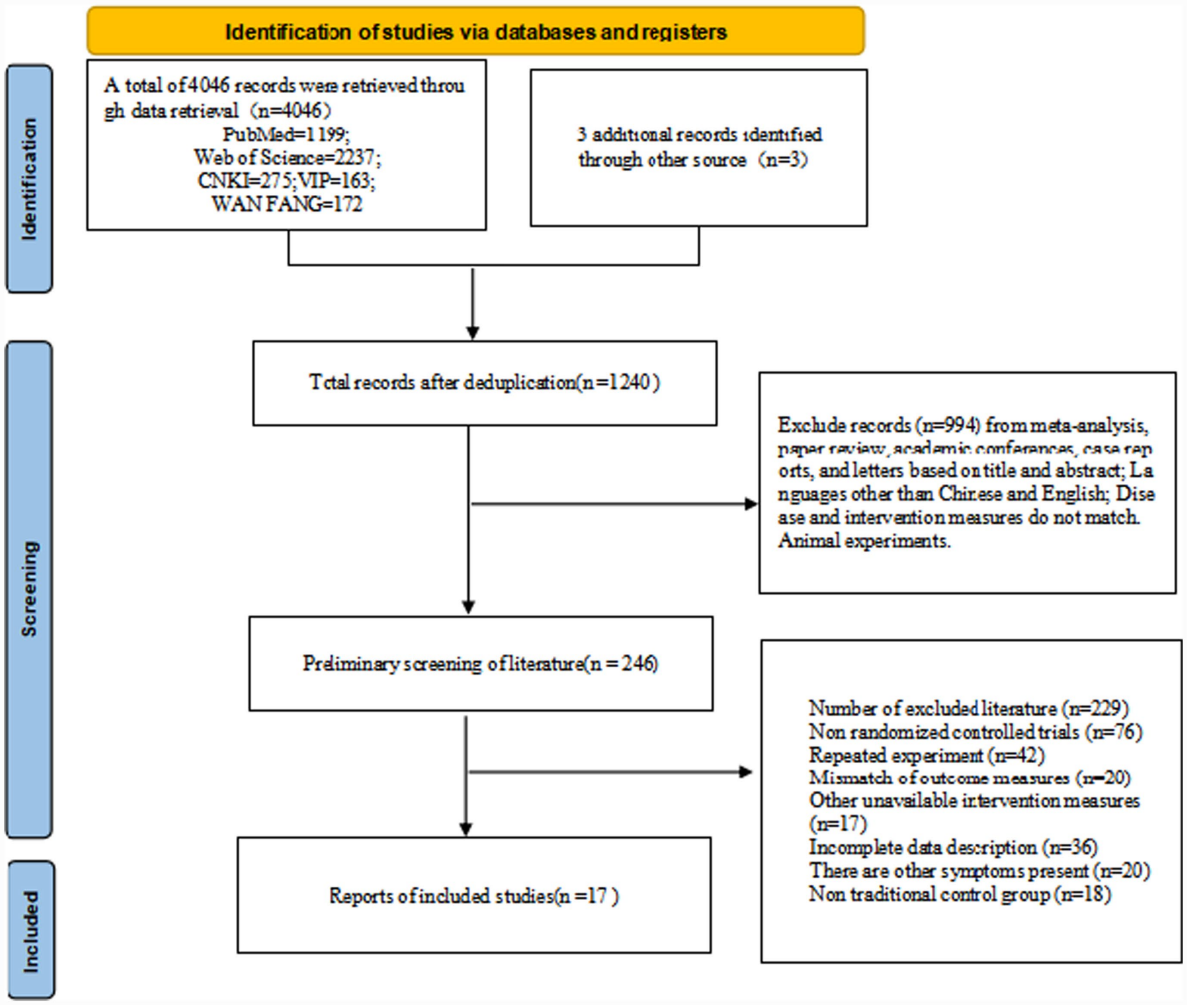
Preferred reporting items for systematic reviews and meta-analyses (PRISMA) flow diagram.

The literature search was conducted strictly according to the protocol outlined in the PRISMA flow diagram (Figure 1). Initial searches across multiple databases yielded 4,049 records. After removing duplicates, 1,240 documents remained for screening. Of these, 994 were excluded based on titles and abstracts (e.g., reviews, conference articles, non-English/Chinese literature, animal experiments, and interventions that did not match the study’s criteria). Subsequently, full-text reviews were conducted on the remaining 246 studies, resulting in the exclusion of 229 articles. Specific reasons for exclusion included: non-RCTs (76), duplicate experiments (42), incomplete data description (36), mismatched outcome indicators (20), participants with other symptoms (20), non-traditional control group settings (18), and other unavailable intervention measures (17). Ultimately, 17 studies met all inclusion criteria and were included in the systematic review and network meta-analysis (see Figure 1).

### 3.2 Characteristics of included studies

The 17 included studies were published between 2011 and 2025 (Table 2). Specifically, there were 5 papers from 2025 (17–21), 3 from 2023 (22–24), 2 each from 2021 (25, 26) and 2022 (27, 28), and 1 each from 2024, 2020, 2019, 2018, and 2011 (29–33). The total sample size was 927, with individual study sample sizes ranging from 22 to 125. The age distribution of subjects showed a bimodal characteristic: a younger group around 20 years old and a young-to-middle-aged group aged 30–54. Regarding physical status, the mean BMI of subjects in the vast majority of studies ranged from approximately 26–39 kg/m^2^, indicating overweight or obese populations, which ensured consistency in metabolic risk characteristics. Regarding intervention frequency, most studies used a training frequency of 3 or 5 times/week, with intervention periods mainly concentrated between 8 and 12 weeks, reaching up to 6 months (30, 31).

**Table 2:** Characteristics of the included studies.

Author year	Age (Mean ± SD)	BMI (kg/m^2^)	Intervention group (sample size)	Control group (sample size)	Intervention frequency	Training intensity (I)	Single training duration (T)	Duration (weeks)	Exercise dosage (h)	Outcome measure
Yu, 2023 (22)	T: 19.91 ± 1.57 C: 20.43 ± 1.93	T: 29.07 ± 2.14 C: 29.44 ± 2.61	AE (*n* = 9)	Wait-list (*n* = 15)	5 times a week	65–75% Maximum heart rate (MHR)	90 min	12	90	PSQI, BMI
Li, 2023 (23)	T: 20.00 ± 0.93 C: 20.00 ± 2.13	T: 28.45 ± 2.67 C: 26.83 ± 2.90	ART (*n* = 20)	Wait-list (*n* = 20)	3 times a week	RT 50–60% 1 repetitions maximum (RM)/AE 50–60% MHR	60 min	8	24	PSQI, BMI
Meng et al., 2025 (17)	T: 20.4 ± 2.18 C: 20.5 ± 2.16	T: 39.06 ± 5.90 C: 38.48 ± 5.34	AE (*n* = 25)	Wait-list (*n* = 20)	5–6 times a week	Heart rate reserve 20–40%	120 min	8	88	PSQI, BMI
Duncan et al. 2020 (30)	T1: Strengthening intervention group 45.4 ± 10.2 T2: Traditional intervention group 47.2 ± 9.4 T3: Merge intervention groups 46.3 ± 9.8 C: 40.5 ± 10.7	T1: Strengthening intervention group 31.9 ± 4.0 T2: Traditional intervention group 31.7 ± 3.9 T3: Merge intervention groups 31.7 ± 3.9 C: 31.4 ± 3.8	ART (*n* = 80)	Wait-list (*n* = 36)	≥2 times/week	Aerobic intensity: >100 mg (accelerometer reading)/self-perceived moderate to vigorous intensity. Resistance intensity: body weight	150 min	24	58.5	PSQI
Mary et al. 2024 (29)	T1: AE 38.6 ± 8.6, T2: RE 34.8 ± 9.5, T3: ART 37.7 ± 6.7 C: 34.1 ± 9.0	T1: AE 39.7 ± 6.0, T2: RE 37.01 ± 7.1, T3: 38.5 ± 6.7 C: 36.7 ± 6.6	AE (*n* = 14) RT (*n* = 11) ART (*n* = 15)	Wait-list (*n* = 16)	3 times a week	AE: 1–2 weeks 40–50% heart rate reserve (HRR) 11–12 weeks 75–80% (HRR) RE: 1–2 weeks 2 × 12 times (40–50% 1 RM) -12 weeks 3–6 groups × 12 times (75–80% 1 RM) ART: consists of two parts, AE and RE, with a ratio of 50:50.	50 min	12	AE: 30 RE: 30 ART: 30 Control: 30	PSQI
Ezpeleta et al., 2023 (24)	T: 44 ± 3 C: 44 ± 3	T: 37 ± 6 C: 37 ± 5	AE (*n* = 15)	Wait list (*n* = 20)	5 times a week	Moderate intensity aerobic exercise, gradually increasing exercise intensity in the first 4 weeks From 65 to 80% (maximum heart rate [HRmax])	60 min	12	60	PSQI
Wilson et al., 2022 (28)	T: 43.7 ± 10 C: 45.6 ± 11.4	T: 28.3 ± 1.7 C: 28.5 ± 3.4	PA (*n* = 67)	Wait list (*n* = 58)	None	Moderate-to-vigorous-intensity exercise	Weekly > 150 min moderate intensity MVPA More than 75 min of intense MVPA/week	16	40	PSQI
Bugday et al., 2025 (18)	T: 47.3 ± 4.5 C: 46.8 ± 3.7	T: Level 1 (30.0%), Level 2 (60.0%), Level 3 (10.0%) C: Level 1 (35.0%), Level 2 (45.0%), Level 3 (20.0%)	RT (*n* = 20)	Calorie-restricted diet (*n* = 20)	3 times a week	60–80% 1 RM	45–60 min	12	30	PSQI
Kirmizigil et al., 2025 (21)	T: 46.7 ± 9.77 C: 45.37 ± 9.63	T: 29.09 ± 2.61 C: 29.0 ± 2.60	Pilates (*n* = 23)	Wait-list (*n* = 24)	3 times a week	1–2 weeks (beginner): Use green and yellow springs, 2 groups, 10 repetitions per group Week 3–6 (Intermediate): Use green and blue springs, 2 sets, 12 repetitions per set Week 7–8 (Advanced): Use green and blue springs, 2 sets, 15 repetitions per set	50–60 min	8	22	PSQI, BMI
Cui et al., 2025 (20)	T: 20.2 ± 0.9 C: 20 ± 1	T: 26.7 ± 1.6 C: 27.3 ± 3.4	RT (*n* = 13)	Regular lifestyle (*n* = 12)	3 times a week	Four groups, 8–12 times per group. 60–70% 1 RM	45 min	8	18	PSQI, BMI
Leonel et al., 2022 (27)	T: 37.44 ± 3.97 C: 34.69 ± 7.45	T: 32.27 ± 2.33 C: 33.19 ± 2.93	ART (*n* = 9)	Wait-list (*n* = 13)	3 times a week	Moderate intensity aerobic: 50–59% heart rate reserve (HRR) Resistance: 2 groups, 10–12 repetitions per group, maximum value (RM)	60 min	16	48	PSQI
Christina et al. 2021 (25)	T: 53.5 ± 10.4 C: 53.5 ± 10.4	33.5 ± 5.5	ART (*n* = 46)	Usual care (*n* = 45)	3 times a week	Aerobic 65–80% detection of heart rate (HR) Resistance, upper limb: 60% 1 RM, lower limb: 80% 1 RM	1. 3 days of aerobic therapy with resistance for 80 min 2 days of aerobic 50 min	16	56	PSQI
Rshikesan et al., 2018 (32)	T: 40.3 ± 8.74 C: 42.2 ± 12.06	T: 28.7 ± 2.35 C: 27.7 ± 2.05	Yoga (*n* = 37)	Walking (*n* = 35)	5 times a week	Warm up 10 min Suryanamaskara 10 min Asana 30 min Pranayama 15 min Meditation 15 min	90 min	14	105	PSQI, BMI
Quist et al., 2019 (31)	T: 32 ± 7 C: 35 ± 7	T: 29.3 ± 2 C: 30.1 ± 2.3	PA (*n* = 28)	Regular lifestyle (*n* = 16)	5 times a week	50% VO_2_ peak-reserve	54 ± 11 min	24	92	PSQI
Kline et al., 2011 (33)	T: 47.6 ± 1.3 C: 45.9 ± 2.2	T: 35.5 ± 1.2 C: 33.6 ± 1.4	PA (*n* = 27)	Flexibility exercises (*n* = 16)	4 times a week/control group 2 times a week	60% heart rate reserve (HRR) Resistance training with 8 different exercises, 2 groups, 10–12 times per group	37.5 min	12	36	PSQI
Yan et al., 2025 (19)	T: 33.6 ± 7.4 C: 38.2 ± 9.3	T: 27.77 (M (IQR): 25.62, 29.62) C: 27.04 (M (IQR): 25.33, 29.23)	Baduanjin (*n* = 26)	Health Education (*n* = 24)	3 times a week	Average heart rate 90–105 beats/min Accounts for 50–60% of maximum heart rate (HRmax)	60 min	12	36	PSQI, BMI
Saidi et al., 2021 (26)	T: 54.7 ± 8.5 C: 53.5 ± 7.5	T: 35.6 ± 6.7 C: 35.6 ± 6.7	PA (*n* = 16)	Evening Exercise (*n* = 12)	3 times a week	Moderate intensity, aerobic exercise: maximum heart rate of 60% Strength training: 60% RM	90 min	12	54	PSQI, BMI

MVPA: Moderate-to-Vigorous Physical Activity.

The exercise intervention protocols adopted by the experimental groups showed significant diversity, covering aerobic exercise, resistance training, mixed training, and mind–body exercises. Some studies utilized a comprehensive mode of combined aerobic and resistance training (ART). For example, Mary et al. (29) implemented a 12-week, 3-times-weekly ART intervention, explicitly setting the aerobic-to-resistance ratio at 50:50 and progressively increasing aerobic intensity. Christina et al. (25) designed a more intensive protocol. Other studies focused on single aerobic exercise or high-intensity physical activity.

Some studies employed specialized equipment or traditional practices. Kirmizigil et al. (21) used Pilates with different-colored springs to distinguish intensity levels. Yan Yu et al. (19) adopted traditional Chinese Baduanjin. Rshikesan et al. (32) implemented a yoga intervention. Pure resistance training was also adopted by some studies (18, 20). All studies measured the Pittsburgh Sleep Quality Index (PSQI) as a core outcome, and nearly half of them (8 studies) also monitored BMI to assess improvements in body composition (see Table 2).

### 3.3 Literature quality assessment

**Figure 2:**
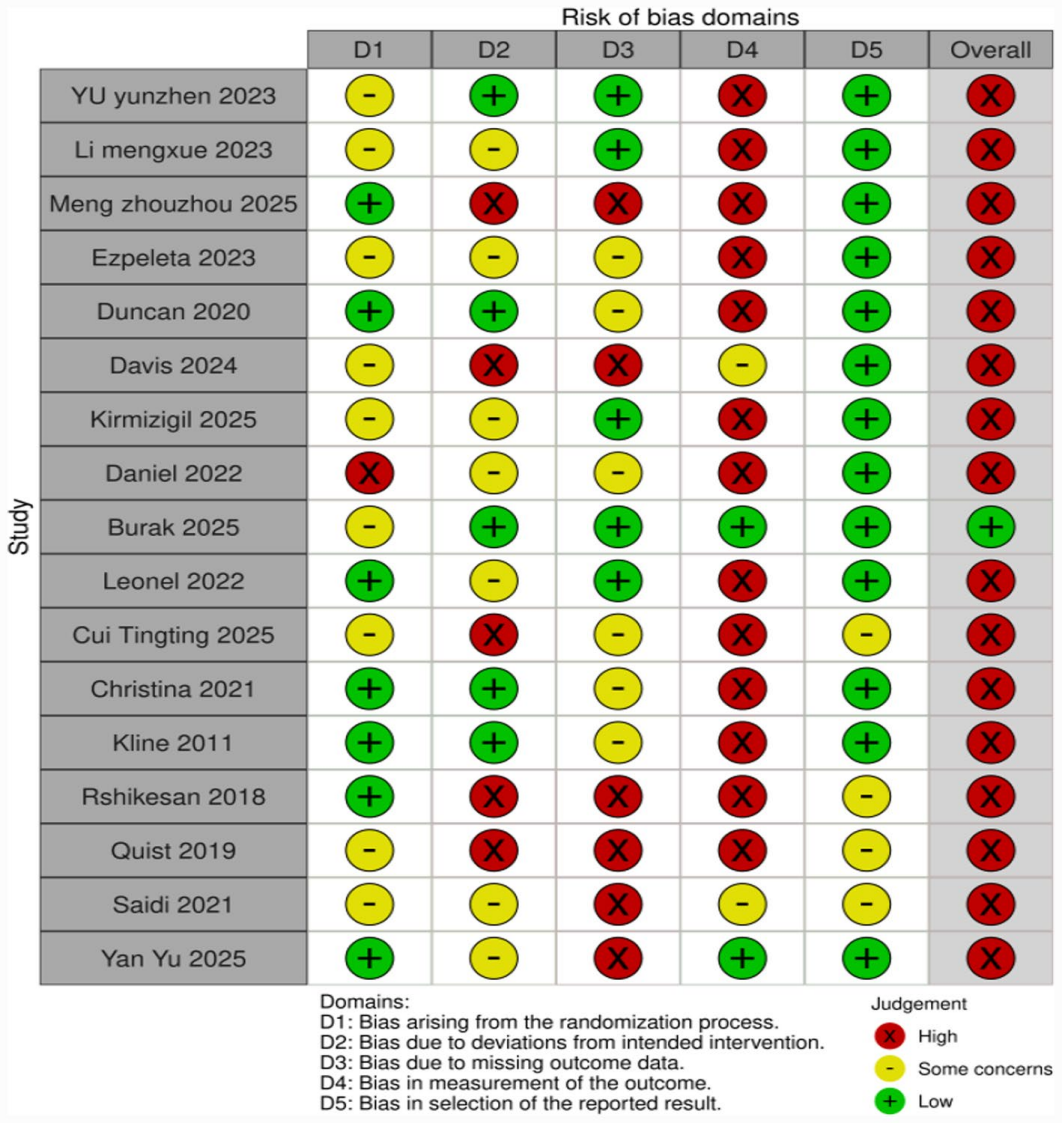
Risk of bias summary: review of the authors’ judgments about each risk of bias item for each included study.

**Figure 3:**
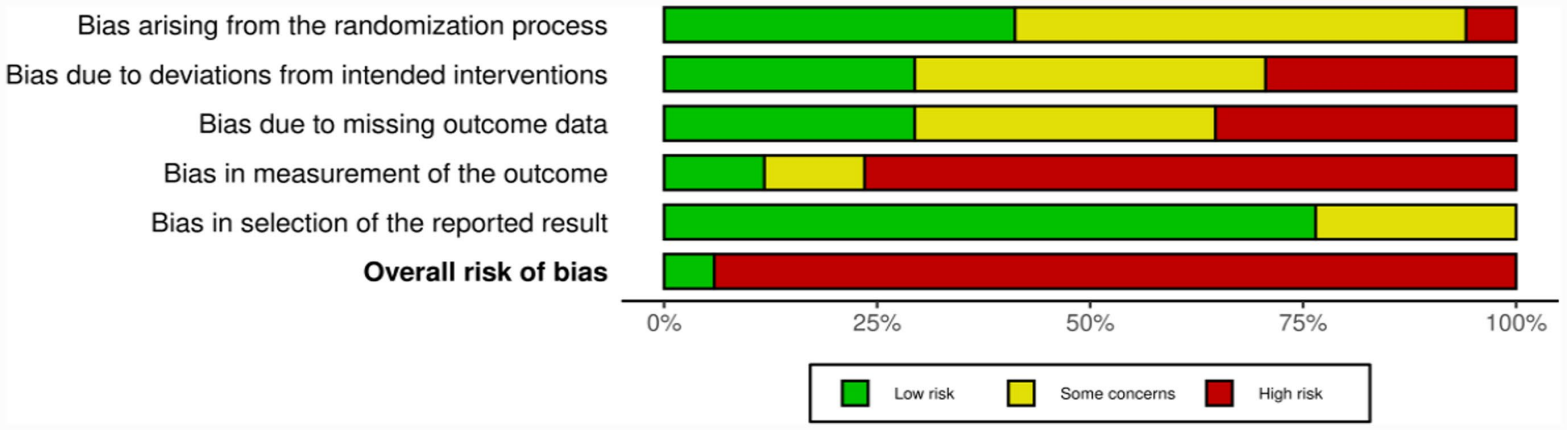
Risk of bias graph: review authors’ judgments about each risk of bias item, presented as percentage of included studies.

We assessed the risk of bias using the Cochrane risk-of-bias (RoB) 2.0 tool (see Figures 2, 3). Overall, there were certain concerns regarding the methodological quality of the included studies. The risk of bias mainly stemmed from the difficulty in blinding exercise interventions (D2: Deviations from intended interventions) and insufficient implementation of blinding for outcome assessors (D4: Measurement of the outcome). This phenomenon may be attributed to the fact that the PSQI is a subjective self-reported outcome, and participants were not blinded. Such lack of blinding is virtually inevitable in exercise intervention studies, potentially leading to expectation bias. In contrast, risks regarding the randomization process (D1), missing data (D3), and selective reporting (D5) were generally low.

### 3.4 Quantitative synthesis methods

Data synthesis was performed using the network meta package in Stata 18.0 to analyze the continuous variables. Due to variations in measurement tools, the standardized mean difference (SMD) was employed to synthesize effect sizes, with the significance level set at *α* = 0.05. To assess the clinical relevance of the findings beyond statistical significance, we incorporated the Minimal Important Difference (MID) analysis alongside the SMD results. Specifically, for the PSQI, a reduction of >3 points (or an SMD of 0.5) is generally considered clinically important (34–37), while for BMI, a reduction of >5% or >1 kg/m^2^ is typically regarded as clinically significant for health outcomes in obese populations (38, 39). To assess network robustness, node analysis techniques were used to detect inconsistencies in closed-loop structures. If the loop inconsistency test yielded *p* > 0.05, a consistency model was applied; local inconsistency was further assessed using the node-splitting method (if *p* < 0.05, traditional meta-analysis was used for direct comparison). Finally, treatment efficacy rankings were determined using the Surface Under the Cumulative RAnking curve (SUCRA) to perform cluster analysis for identifying optimal treatment combinations, and potential publication bias was examined using adjusted comparison funnel plots and Egger’s regression test.

### 3.5 Sleep quality outcome analysis

#### 3.5.1 Network plot of included studies

**Figure 4:**
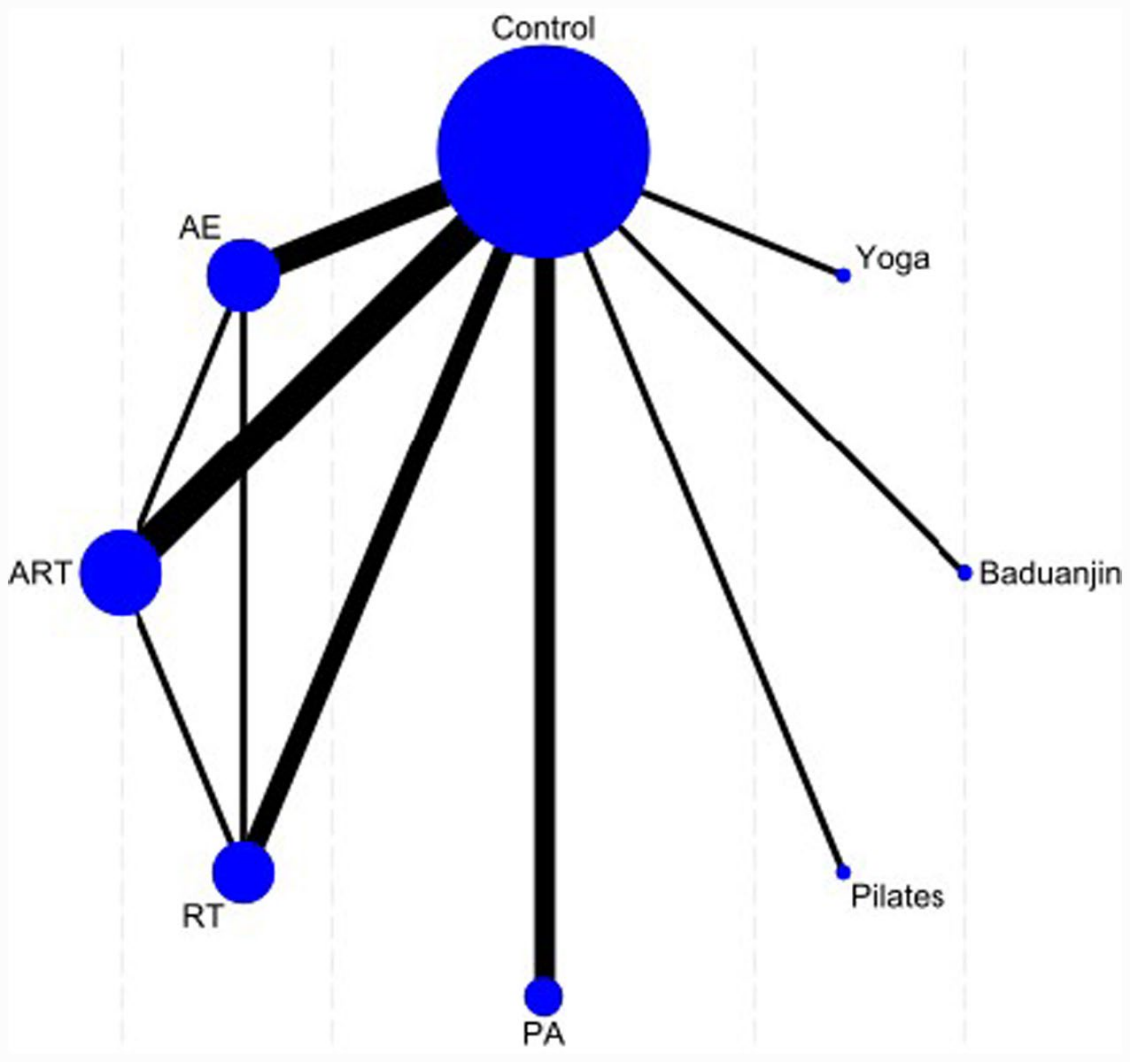
Network plot of sleep quality.

In Figure 4, the eight nodes represent the seven intervention measures, and the connecting lines between the nodes indicate direct comparisons. The intervention groups include resistance training (RT), combined aerobic and resistance training (ART), Baduanjin, yoga, Pilates, physical activity (PA), and aerobic exercise (AE), while the control group consists of a waiting list or routine care. In the network diagram, the width of the connecting lines reflects the frequency of direct comparisons between pairs of interventions.

Seventeen studies evaluated sleep quality and were included in the network meta-analysis (*n* = 921 participants, 7 treatment methods). The figure displays the network plot of eligible comparisons for sleep quality, where most interventions were compared with the control group. The seven interventions were predominantly exercise-based, including Resistance Training (RT), combined aerobic and resistance training (ART), Baduanjin, yoga, Pilates, physical activity (PA), and aerobic exercise (AE).

The global inconsistency test (*χ*^2^ = 1.17, *p* = 0.76) indicated favorable consistency across the included studies. Furthermore, the node-splitting method revealed no significant local inconsistency (all *p* > 0.05), suggesting no substantial discrepancies between direct and indirect evidence across comparisons. For specific closed loops, the *p*-values for key nodes were 0.471 for AE vs. ART, 0.962 for RT vs. AE, and 0.414 for ART vs. RT, respectively. These findings underscore the robustness of the network structure and the reliability of the consistency model employed in this analysis.

#### 3.5.2 Intervention ranking

**Figure 5:**
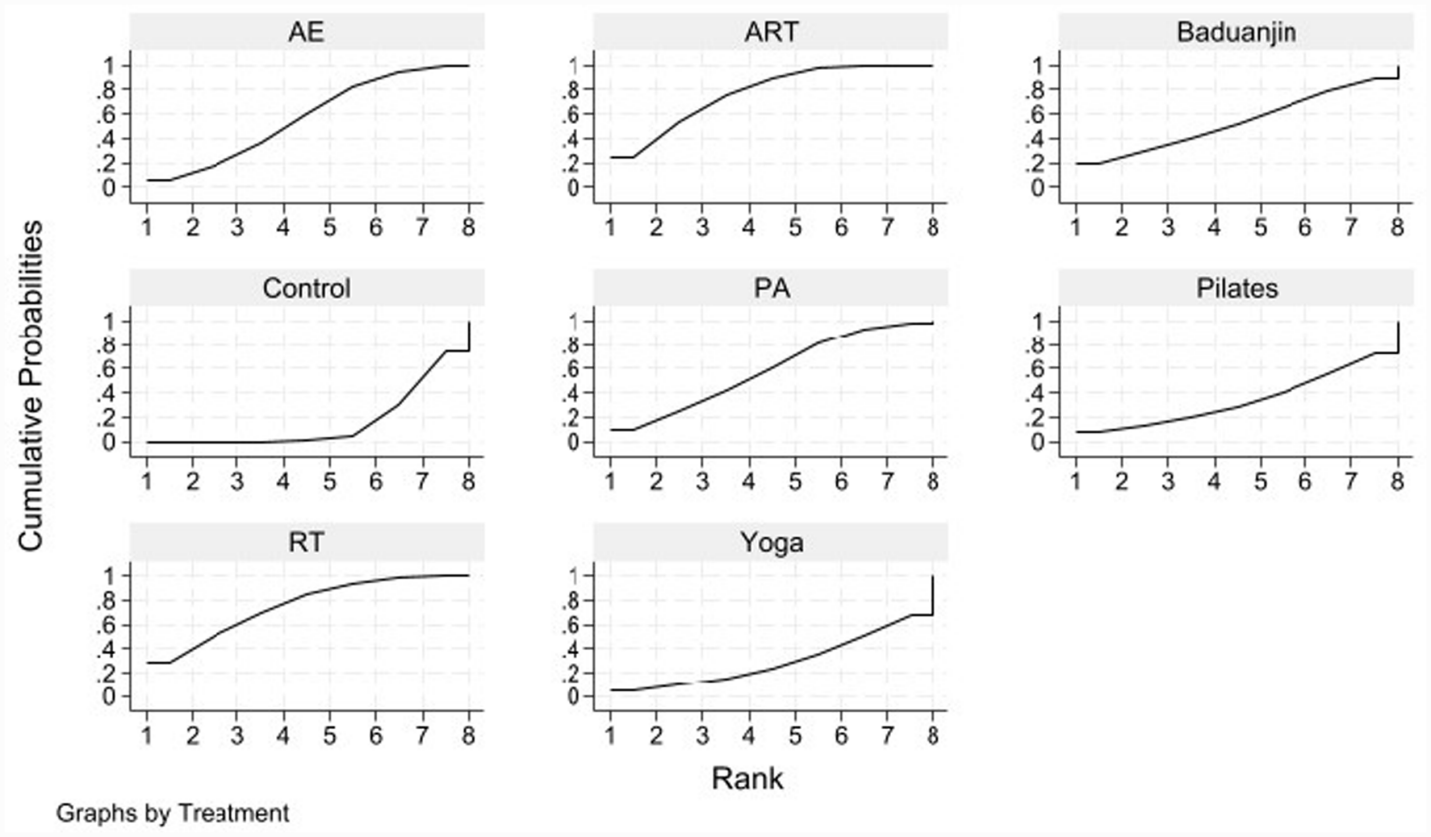
Rank probability plots for sleep outcomes.

We used rank probabilities (Table 3; Figure 5) to assess the likelihood of each intervention being the best option. The results showed that combined aerobic and resistance training (ART) had the highest probability (SUCRA ≈ 77.1%) of being the optimal intervention for improving sleep quality. This was followed by resistance training (RT) (SUCRA ≈ 75.2%), which also demonstrated robust intervention effects. Three interventions—physical activity (PA) (SUCRA ≈ 58.5%), aerobic exercise (AE) (SUCRA ≈ 56.7%), and Baduanjin (SUCRA ≈ 53.6%)—were superior to the control group in improving sleep quality, but their efficacy was lower than that of ART and RT. The remaining interventions, such as yoga and Pilates, exhibited milder effects on sleep treatment.

**Table 3:** SUCRA values for sleep quality outcomes.

Treatment	SUCRA	Probability of being the best (Prbest)	Mean rank
AE	56.7	6.2	4.0
ART	77.1	24.6	2.6
Baduanjin	53.6	18.8	4.2
Control	15.8	0.0	6.9
PA	58.5	10.0	3.9
Pilates	33.6	7.1	5.6
RT	75.2	28.0	2.7
Yoga	29.5	5.2	5.9

#### 3.5.3 League table analysis of interventions

Table 4 presents the league table of relative effects, illustrating the efficacy of all interventions compared with the conventional control (CON). The results indicated that, compared with the conventional control, all seven active exercise interventions demonstrated statistically significant improvements in sleep quality (as measured by Pittsburgh Sleep Quality Index, PSQI, scores).

**Table 4:** League table of sleep outcomes.

Yoga	RT	Pilates	PA	Control	Baduanjin	ART	AE
Yoga	−0.85 (−2.35, 0.66)	−0.10 (−1.95, 1.76)	−0.56 (−2.06, 0.94)	0.10 (−1.19, 1.39)	−0.50 (−2.36, 1.35)	−0.84 (−2.26, 0.57)	−0.54 (−1.99, 0.91)
0.85 (−0.66, 2.35)	RT	0.75 (−0.79, 2.30)	0.29 (−0.80, 1.38)	0.95 (0.17, 1.73)	0.34 (−1.20, 1.89)	0.01 (−0.91, 0.92)	0.31 (−0.65, 1.26)
0.10 (−1.76, 1.95)	−0.75 (−2.30, 0.79)	Pilates	−0.46 (−2.00, 1.07)	0.19 (−1.14, 1.53)	−0.41 (−2.29, 1.48)	−0.75 (−2.20, 0.71)	−0.44 (−1.93, 1.04)
0.56 (−0.94, 2.06)	−0.29 (−1.38, 0.80)	0.46 (−1.07, 2.00)	PA	0.66 (−0.11, 1.42)	0.05 (−1.48, 1.59)	−0.28 (−1.25, 0.68)	0.02 (−0.99, 1.03)
−0.10 (−1.39, 1.19)	−0.95 (−1.73, -0.17)	−0.19 (−1.53, 1.14)	−0.66 (−1.42, 0.11)	Control	−0.60 (−1.93, 0.73)	−0.94 (−1.53, -0.35)	−0.64 (−1.30, 0.02)
0.50 (−1.35, 2.36)	−0.34 (−1.89, 1.20)	0.41 (−1.48, 2.29)	−0.05 (−1.59, 1.48)	0.60 (−0.73, 1.93)	Baduanjin	−0.34 (−1.79, 1.12)	−0.04 (−1.52, 1.45)
0.84 (−0.57, 2.26)	−0.01 (−0.92, 0.91)	0.75 (−0.71, 2.20)	0.28 (−0.68, 1.25)	0.94 (0.35, 1.53)	0.34 (−1.12, 1.79)	ART	0.30 (−0.53, 1.14)
0.54 (−0.91, 1.99)	−0.31 (−1.26, 0.65)	0.44 (−1.04, 1.93)	−0.02 (−1.03, 0.99)	0.64 (−0.02, 1.30)	0.04 (−1.45, 1.52)	−0.30 (−1.14, 0.53)	AE

Specifically, resistance training (RT) exhibited the most favorable mean effect size (SMD = −0.95, 95% CI [−1.73, −0.17]), indicating the most significant effect on improving sleep. Combined aerobic and resistance training (ART) (SMD = −0.94, 95% CI [−1.53, −0.35]) likewise demonstrated a robust effect on sleep improvement. Aerobic exercise (AE) (SMD = −0.64, 95% CI [−1.30, 0.02]) showed a trend of superiority over the remaining exercise interventions, although the difference was not statistically significant. Yoga, Pilates, Baduanjin, and physical activity (PA) had more modest impacts on sleep quality improvement; their 95% confidence intervals crossed the null value of 0, indicating a lack of statistical significance.

### 3.6 BMI analysis

#### 3.6.1 Network plot of included studies

**Figure 6:**
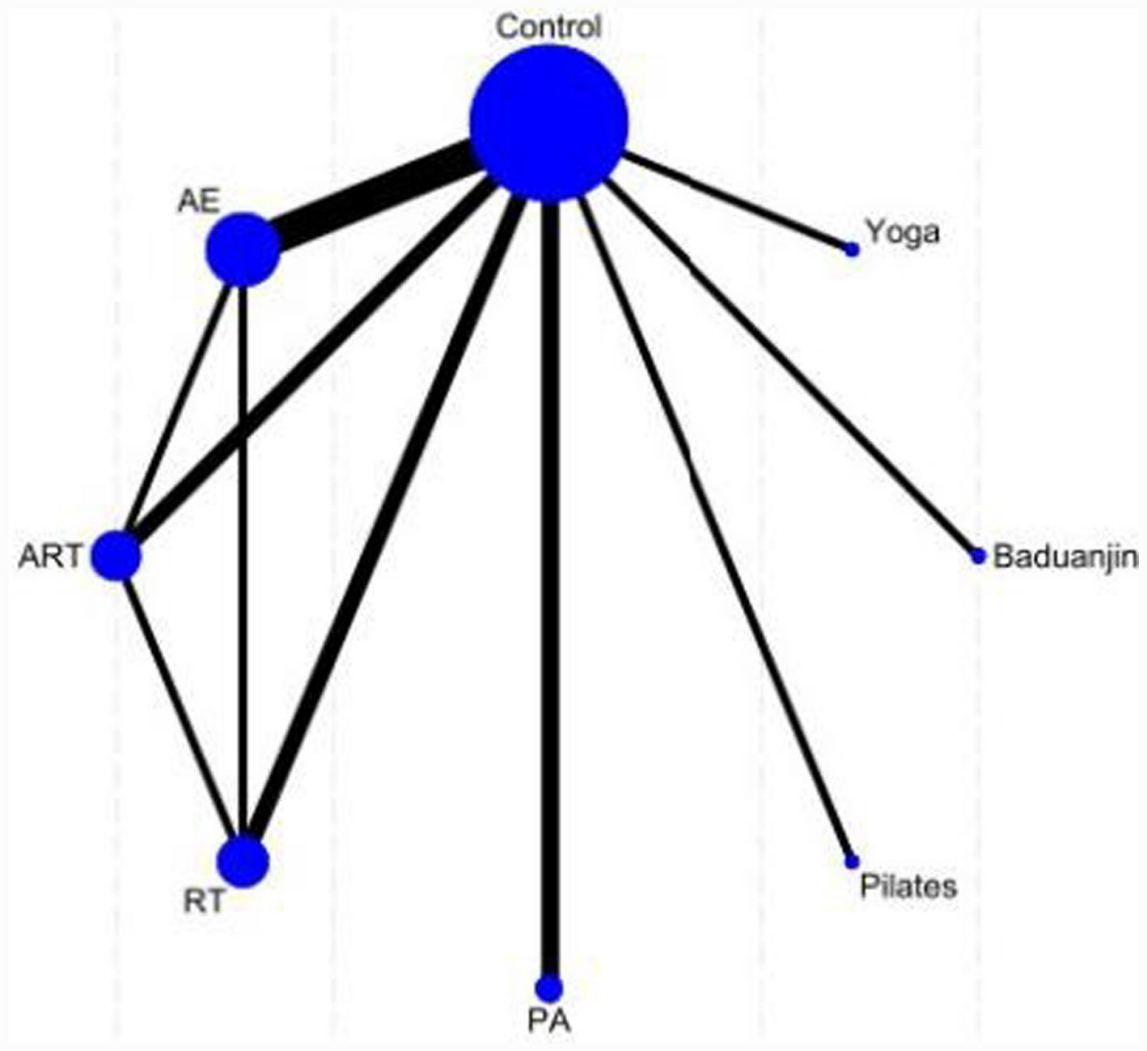
Network plot of BMI.

In Figure 6, the eight nodes represent the seven intervention measures, and the connecting lines between the nodes indicate direct comparisons. The intervention groups include resistance training (RT), combined aerobic and resistance training (ART), Baduanjin, yoga, Pilates, physical activity (PA), and aerobic exercise (AE), while the control group consists of a waiting list or routine care. In the network diagram, the width of the connecting lines reflects the frequency of direct comparisons between pairs of interventions.

Twelve studies evaluating BMI were included in the network meta-analysis (*n* = 614 participants, 8 intervention methods). The figure displays the network plot of eligible comparisons for BMI, with most interventions compared against routine care (Control) or sham intervention (AE). Among the 30 intervention measures, psychological–behavioral and exercise interventions were predominant (*n* = 24), including resistance training (RT), combined aerobic and resistance training (ART), Baduanjin, yoga, Pilates, and physical activity (PA).

The global inconsistency test (*χ*^2^ = 0.64, *p* = 0.8868) indicated favorable consistency across the included studies. Furthermore, the node-splitting method revealed no significant local inconsistency (all *p* > 0.05), suggesting no substantial discrepancies between direct and indirect evidence across comparisons. For specific closed loops, the *p*-values for key nodes were 0.416 for AE vs. ART, 0.401 for RT vs. AE, and 0.971 for ART vs. RT, respectively. These findings underscore the robustness of the network structure and the reliability of the consistency model employed in this analysis.

#### 3.6.2 Intervention ranking

**Figure 7:**
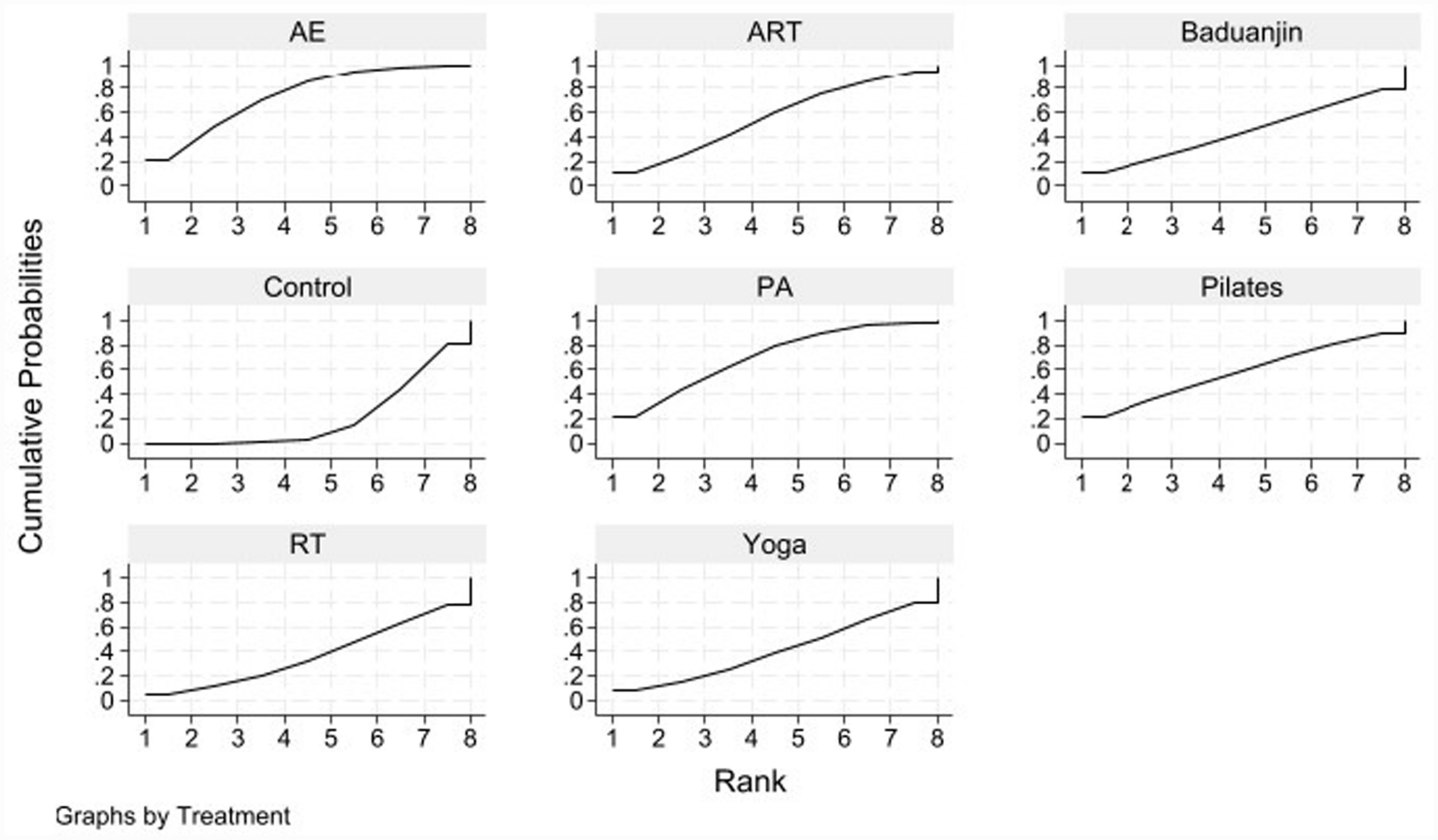
Rank probability plots for BMI.

We used rank probabilities (Table 5; Figure 7) to assess the likelihood of each intervention being the best option. The results indicated that aerobic exercise (AE) had the highest probability (SUCRA ≈ 74.0%) of being the optimal intervention for improving BMI. This was followed by physical activity (PA) (SUCRA ≈ 70.3%), Pilates (SUCRA ≈ 57.7%), and combined aerobic and resistance training (ART) (SUCRA ≈ 56.2%). While these three interventions demonstrated superior efficacy in improving BMI compared to the control group, they were less effective than aerobic exercise. The remaining interventions, such as yoga and Baduanjin, exhibited more modest effects on BMI improvement.

**Table 5:** SUCRA values for BMI.

Treatment	SUCRA	Prbest	Mean rank
AE	74.0	21.7	2.8
ART	56.2	11.5	4.1
Baduanjin	44.3	10.9	4.9
Control	20.3	0.0	6.6
PA	70.3	22.0	3.1
Pilates	57.7	21.5	4.0
RT	36.6	4.8	5.4
Yoga	40.4	7.6	5.2

#### 3.6.3 League table analysis of interventions

Table 6 displays the league table of relative effects, illustrating the efficacy of all interventions compared with the conventional control (CON). The results indicated that, compared to the routine control, all 7 active exercise interventions demonstrated statistically significant advantages in improving BMI. Specifically, aerobic exercise (AE) displayed the best mean effect size (SMD = −0.43, 95% CI [−0.80, −0.05]), indicating the most significant effect on improving BMI. Interventions such as combined aerobic and resistance training (ART), resistance training (RT), physical activity (PA), yoga, and Pilates showed favorable effects on BMI improvement compared to the control group; however, their 95% confidence intervals crossed the null value of 0, indicating no statistical significance.

**Table 6:** League table of BMI.

Yoga	RT	Pilates	PA	Control	Baduanjin	ART	AE
Yoga	0.02 (−0.83, 0.88)	−0.18 (−1.12, 0.77)	−0.28 (−1.05, 0.50)	0.12 (−0.50, 0.75)	−0.04 (−0.98, 0.89)	−0.15 (−0.96, 0.66)	−0.30 (−1.03, 0.43)
−0.02 (−0.88, 0.83)	RT	−0.20 (−1.12, 0.72)	−0.30 (−1.05, 0.44)	0.10 (−0.48, 0.69)	−0.07 (−0.97, 0.84)	−0.17 (−0.88, 0.53)	−0.33 (−0.97, 0.32)
0.18 (−0.77, 1.12)	0.20 (−0.72, 1.12)	Pilates	−0.10 (−0.95, 0.75)	0.30 (−0.41, 1.01)	0.14 (−0.86, 1.13)	0.03 (−0.85, 0.91)	−0.12 (−0.93, 0.68)
0.28 (−0.50, 1.05)	0.30 (−0.44, 1.05)	0.10 (−0.75, 0.95)	PA	0.40 (−0.06, 0.87)	0.24 (−0.60, 1.07)	0.13 (−0.57, 0.82)	−0.02 (−0.61, 0.56)
−0.12 (−0.75, 0.50)	−0.10 (−0.69, 0.48)	−0.30 (−1.01, 0.41)	−0.40 (−0.87, 0.06)	Control	−0.17 (−0.86, 0.53)	−0.28 (−0.79, 0.24)	−0.43 (−0.80, -0.05)
0.04 (−0.89, 0.98)	0.07 (−0.84, 0.97)	−0.14 (−1.13, 0.86)	−0.24 (−1.07, 0.60)	0.17 (−0.53, 0.86)	Baduanjin	−0.11 (−0.98, 0.76)	−0.26 (−1.05, 0.53)
0.15 (−0.66, 0.96)	0.17 (−0.53, 0.88)	−0.03 (−0.91, 0.85)	−0.13 (−0.82, 0.57)	0.28 (−0.24, 0.79)	0.11 (−0.76, 0.98)	ART	−0.15 (−0.74, 0.44)
0.30 (−0.43, 1.03)	0.33 (−0.32, 0.97)	0.12 (−0.68, 0.93)	0.02 (−0.56, 0.61)	0.43 (0.05, 0.80)	0.26 (−0.53, 1.05)	0.15 (−0.44, 0.74)	AE

### 3.7 Dose–response analysis

#### 3.7.1 Sleep dose–response analysis

**Figure 8:**
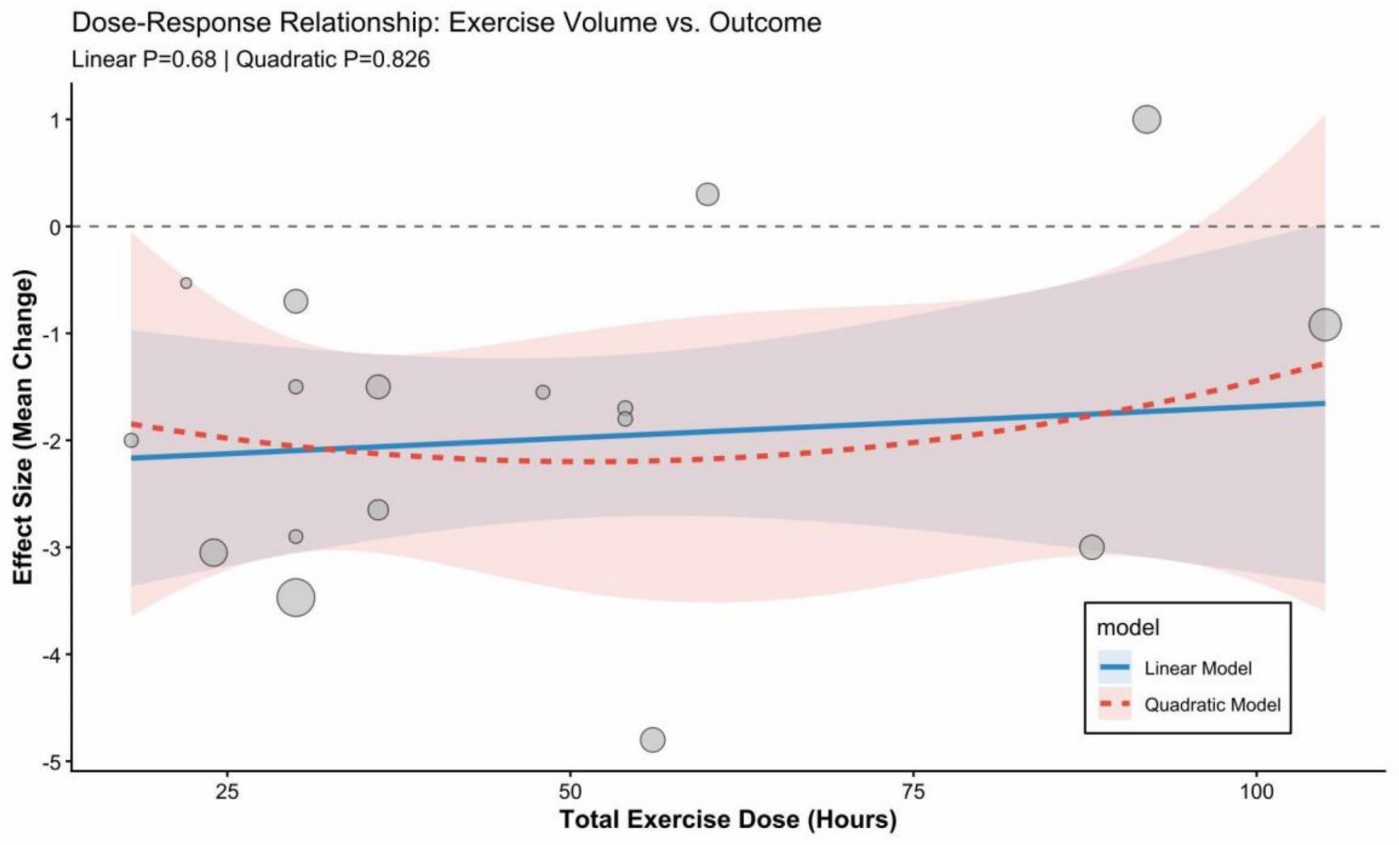
Meta-regression of total exercise dose and improvement in sleep quality.

Meta-regression analysis revealed that total exercise dosage did not significantly moderate intervention efficacy (*p* > 0.05). As illustrated in Figure 8, within the analyzed dosage range (18–105 h), meta-regression did not detect a significant linear (*p* = 0.68) or non-linear (p_quadratic = 0.826) dose–response relationship between exercise dosage and the outcome measures.

#### 3.7.2 BMI dose–response analysis

**Figure 9:**
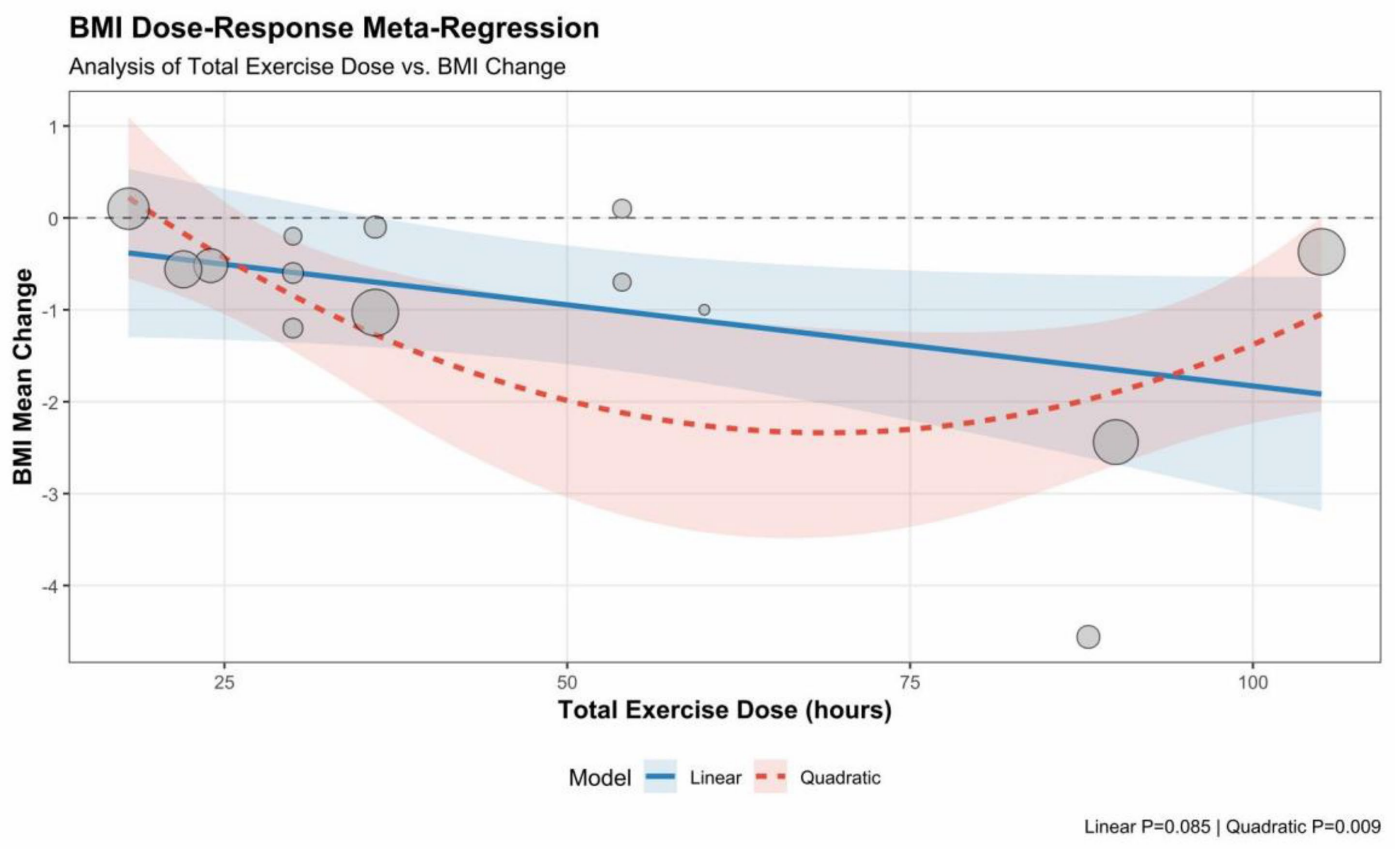
Meta-regression of total exercise dose and improvement in BMI.

**Figure 10:**
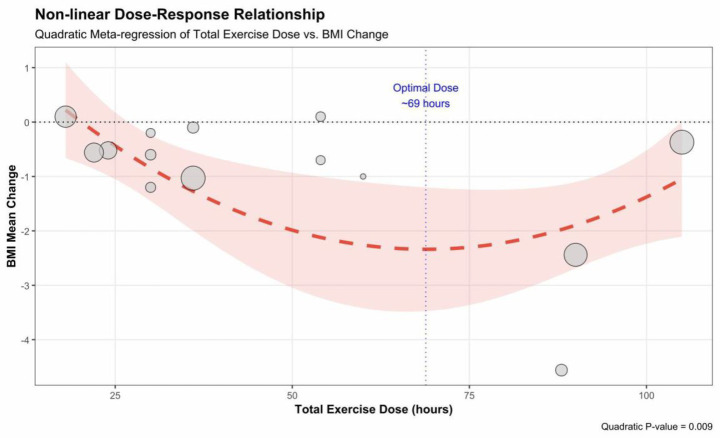
Non-linear dose–response relationship between total exercise dose and improvement in BMI.

Meta-regression analysis revealed a significant non-linear dose–response relationship between total exercise dosage and BMI improvement (p_quadratic = 0.009), whereas the linear trend did not reach statistical significance (p_linear = 0.085). As shown in the figure, the fitted curve presents a typical “U-shaped” trend: as the total exercise dosage increases (from 20 to 60 h), the magnitude of BMI reduction increases significantly; however, when the total dosage exceeds approximately 70 h, these marginal returns begin to diminish, and no further significant improvement is observed. This indicates that a non-linear dose–response relationship may underlie the data, necessitating verification through a quadratic regression model (Figures 9, 10).

Meta-regression analysis demonstrated a significant non-linear (U-shaped) dose–response relationship between total exercise dosage and BMI improvement (p_quadratic = 0.009), whereas the simple linear trend did not reach statistical significance (*p* = 0.085). The fitted curve suggests the existence of an optimal dosage interval for this intervention mode: as the total exercise dosage increases, the magnitude of BMI reduction initially increases significantly, reaching peak efficacy at a cumulative duration of approximately 60–70 h during the intervention period. However, when the total dosage was increased beyond 90 h, superior weight-loss effects were not observed; instead, a distinct trend of diminishing marginal returns, or even a rebound, was evident.

### 3.8 Quality of evidence

Based on the GRADE evaluation system, the overall quality of the evidence included in this study ranged from low to moderate levels (see Table 7). The quality of evidence for Sleep Quality was rated as “moderate” (17 studies, 921 participants). This outcome was downgraded due to inherent methodological limitations in the included studies (downgrade a). Specifically, according to the RoB 2.0 tool assessment, due to the specific nature of exercise interventions, blinding participants and personnel was difficult in the vast majority of included studies, leading to a High Risk of bias in the domains of “deviations from intended interventions (D2)” and “measurement of the outcome (D4).” Nevertheless, this outcome did not exhibit serious inconsistency or imprecision, and the effect sizes of the primary interventions were statistically significant.

**Table 7:** Grade assessment of quality of evidence.

Outcome measure	Number of studies (Number of participants)	Risk of bias	Heterogeneity	Indirectness	Imprecision	Publication bias	Certainty
PSQI	17 (921)	Downgrade a	Not serious	Not serious	Not serious	Not assessed	⊕ ⊕ ⊕ ⊝ Moderate
BMI	12 (614)	Downgrade a	Not serious	Not serious	Downgrade c	Not assessed	⊕ ⊕ ⊝ ⊝ Low

The quality of evidence for body mass index (BMI) was rated as “low” (12 studies, 614 participants). This outcome was subject to a double downgrade due to High Risk of bias (downgrade a) and imprecision (downgrade c). First, similar to the sleep quality outcome, the risk of bias primarily stemmed from the lack of blinding. Second, regarding precision, although aerobic exercise (AE) demonstrated significant improvement effects, the 95% confidence intervals for the effect sizes of several other interventions (e.g., resistance training, ART, and physical activity) compared to the control group crossed the null value of 0, indicating imprecision in the results. Furthermore, the number of RCTs included in this study permitted an assessment of publication bias; although no obvious publication bias was detected, given that the overall quality of evidence ranges from low to moderate, conclusions regarding the relative effectiveness of different exercise modes should still be interpreted with caution in the context of clinical practice.

## 4 Discussion

### 4.1 Summary of findings

Through this network meta-analysis of exercise on sleep quality in obese populations, we systematically evaluated the effects of seven distinct exercise intervention modes on sleep quality and BMI in overweight and obese populations. The findings revealed that the included active exercise interventions were generally superior to blank control groups in improving the Pittsburgh Sleep Quality Index (PSQI) scores. Regarding the ranking of relative effectiveness, combined aerobic and resistance training (ART) demonstrated the highest potential for improvement (SUCRA ≈ 77.1%), followed by resistance training (RT) (SUCRA ≈ 75.2%), which also exhibited robust improvement effects. Physical activity (PA) (SUCRA ≈ 58.5%) showed moderate-to-strong improvement effects. In terms of improving body composition, aerobic exercise (AE) demonstrated the optimal improvement effect (SUCRA ≈ 74.0%), followed closely by physical activity (SUCRA ≈ 70.3%). Dose–response analysis further suggested potential strategic differences in improving different outcome measures: improvements in sleep quality appeared to possess a potential “low dose, high benefit” characteristic. Meta-regression analysis found no evidence of significant fluctuations in intervention effects with increasing total exercise volume, implying that participants may achieve benefits comparable to those of high cumulative dosages by completing a relatively low cumulative dosage within the intervention period. Conversely, regarding BMI improvement, the data presented a potential non-linear dose–response trend. This suggests that a cumulative total exercise volume of 60–70 h over the entire intervention period (approximately 5–6 h/week for 12 weeks) may be the optimal intervention interval for weight loss. In contrast, excessive training dosages exceeding 90 h within the intervention period may carry the risk of diminishing marginal returns. In summary, different exercise modes have distinct focuses in improving health outcomes for obese populations. Resistance and combined training are most significant for enhancing sleep quality and possess “low dose, high benefit” characteristics. In contrast, aerobic exercise offers superior advantages in reducing BMI, with optimal effects observed at a cumulative intervention duration of 60–70 h.

### 4.2 Comparison with previous studies and mechanism analysis

Our findings demonstrate both consistency with existing literature and unique academic value. In the context of exercise interventions for sleep enhancement, Lin et al. (9) suggested that combined exercise modes hold significant promise by integrating the synergistic benefits of combined aerobic and resistance training. The results of our network meta-analysis (NMA) further quantify this perspective: combined aerobic and resistance training (ART, SUCRA ≈ 77.1%) and Resistance Training alone (RT, SUCRA ≈ 75.2%) demonstrated significant advantages in improving the Pittsburgh Sleep Quality Index (PSQI). Simultaneously, an intriguing asynchrony was identified: while aerobic exercise (AE) maintained an absolute advantage in improving body composition (BMI) (SUCRA ≈ 74.0%), its efficacy in improving sleep quality ranked only fourth (SUCRA ≈ 56.7%). To further explore this discrepancy, we conducted a correlation analysis between the effect sizes of BMI reduction and PSQI improvement across the included studies. The results showed no significant linear correlation (*p* > 0.05). This finding deviates from the traditional assumption that weight loss is the primary driver of sleep improvement (40), implying that while weight reduction remains vital for obese populations, the inclusion of resistance elements may further enhance sleep through weight-independent physiological mechanisms. Furthermore, while resistance training is typically prioritized in older adult populations with sarcopenia (41–43), this study supports its potential applicability in young and middle-aged obese cohorts. Conversely, the lower ranking of mind–body exercises such as Pilates (SUCRA ≈ 33.6%) and yoga (SUCRA ≈ 29.5%) may inversely corroborate that metabolic and physiological expenditure are perhaps more critical than psychological regulation alone in managing obesity-related sleep disorders.

It must be explicitly emphasized that, as this study did not directly assess objective physiological or biochemical markers, the following discussion of mechanisms is primarily based on indirect inferences and theoretical extrapolations from previous literature, rather than definitive empirical conclusions. We propose that the mechanisms by which exercise improves sleep involve two potentially complementary dimensions: weight-dependent and weight-independent pathways. The weight-dependent pathway, largely associated with AE, suggests that reducing BMI may promote upper airway activation and reduce mechanical obstruction by decreasing neck fat deposition (44–46). More critically, the weight-independent pathway may explain why ART and RT achieved superior sleep improvements despite sub-optimal weight loss. In the absence of a significant BMI–sleep correlation, it is plausible that exercise improves sleep through alternative pathways, such as the modulation of the autonomic nervous system (e.g., increased vagal tone), reduction of systemic inflammation (e.g., interleukin 6 [IL-6] and tumor necrosis factor alpha [TNF-α]), or entrainment of circadian rhythms. From a neuromuscular perspective, the moderate-to-high intensity resistance loads (60–80% 1RM) identified in the included literature (18, 20) might induce deep muscle fatigue and Excess Post-exercise Oxygen Consumption (EPOC), thereby increasing sleep pressure and prompting the body to restore homeostasis through increased slow-wave sleep (47, 48). This neural regulation pathway appears to be keenly captured by the PSQI (49). Additionally, RT may improve insulin sensitivity and glucose homeostasis, whereas the ART mode may exert a superposition effect by combining autonomic regulation with skeletal muscle endocrine stimulation (50, 51).

In summary, the cumulative effect of these multiple mechanisms likely provides the biological foundation for ART as a highly effective solution for sleep management in obese populations. Furthermore, the lack of a linear dose–response relationship in our analysis implies that lower doses of exercise may be as effective as higher doses within the studied range. This finding has significant clinical implications, suggesting that patients may not necessarily need to adhere to high-volume training to achieve meaningful sleep benefits, which could improve long-term adherence to exercise prescriptions. This may also explain why some patients perceive improvements in sleep quality through specific exercise modes during the early stages when body weight has not yet significantly decreased (52, 53). By identifying potential weight-independent pathways and the efficacy of moderate exercise volumes, our study provides a more flexible and evidence-based framework for clinical exercise interventions targeting sleep in individuals with obesity.

### 4.3 Practical implications

Beyond statistical significance, the clinical relevance of these interventions warrants attention. In our analysis, combined aerobic and resistance training (ART) and resistance training (RT) demonstrated large effect sizes (SMD of −0.94 and −0.95, respectively), exceeding the threshold for the Minimal Important Difference (MID, typically SMD ≈ 0.5) for sleep quality. Based on these observed benefits, it is suggested that clinical exercise prescriptions be tailored to the patients primary goals. For patients prioritizing sleep improvement, ART and RT may be considered the preferred modalities. Notably, our dose–response analysis indicated no significant linear association between total exercise volume and sleep improvement, implying that participants might achieve meaningful benefits with a moderate dosage rather than necessarily pursuing prolonged cumulative training. In contrast, for patients whose core objective is BMI reduction, aerobic exercise (AE) stands out as an efficient choice, showing moderate-to-large effect sizes that align with clinically recommended weight-loss targets. Crucially, we identified a significant U-shaped non-linear relationship between BMI improvement and exercise volume. To optimize weight-loss benefits, it may be advisable to maintain a cumulative volume of approximately 60–70 h (roughly 5–6 h/week for 12 weeks). Clinicians are advised to inform patients that training volumes exceeding 90 h could potentially carry a risk of diminishing marginal returns.

From a public health and policy perspective, these findings offer an evidence base for updating health management guidelines. It is recommended that governments and public health institutions consider refining the traditional paradigm that emphasizes only aerobic exercise by advocating for the inclusion of resistance and combined training into recommendation categories. Furthermore, public health education strategies could focus on promoting scientific dose–response concepts, emphasizing that favorable outcomes may rely on selecting specific exercise modes and adhering to scientific dosage ranges—thereby helping to mitigate the vicious cycle of “obesity–sleep disorders.”

### 4.4 Study limitations

The primary strength of this study lies in its inaugural application of network meta-analysis to directly compare seven distinct exercise intervention modes—including combined aerobic and resistance training, resistance training, and physical activity—within a unified framework, effectively addressing the limitations of previous single-type comparisons. However, this study is subject to certain limitations. First, regarding methodological quality, achieving double-blinding was difficult due to the nature of exercise interventions, leading to a higher risk of bias in outcome measurement. Second, the reliance on the subjective Pittsburgh Sleep Quality Index (PSQI) may introduce recall bias, as it lacks corroboration from objective physiological indicators. Third, a notable limitation in the dose–response analysis is the heterogeneity of exercise intensity. Our dose calculation formula (Total Dosage = W × F × Duration) primarily quantifies training “volume” but fails to fully account for physiological ‘load’ variations across modalities (e.g., the metabolic equivalent of yoga differs significantly from high-intensity interval training [HIIT]). Although we categorized interventions to mitigate this, the simplified time-based dosage calculation may lead to overgeneralization. Finally, the exploratory analysis of dose–response effects was constrained by the scarcity of data points in the high-dosage range (e.g., cumulative >90 h). This introduces statistical uncertainty into the estimation of the optimal dosage window, and the relevant conclusions require verification with additional empirical data. To address these shortcomings, we restricted inclusion to RCTs and verified robustness of the results using node-splitting and consistency tests.

Future research should focus on addressing these limitations to advance clinical practice. First, incorporating objective sleep measurement tools, such as polysomnography (PSG) or actigraphy, is recommended to validate subjective PSQI findings and reduce measurement bias. Second, more high-quality, large-sample randomized controlled trials are needed to strengthen the direct comparison evidence for superior interventions like combined aerobic and resistance training. Furthermore, there is a critical need to refine quantification standards for exercise load and systematically explore the interaction between different exercise intensities and cumulative dosages, as well as long-term adherence. This will provide clinical practice with more robust and precise evidence-based grounds regarding the “dose–response” relationship.

## 5 Conclusion

This systematic review and network meta-analysis, encompassing 17 randomized controlled trials with 921 participants, provides the first unified framework to evaluate the relative effects of seven exercise modalities on sleep quality and body composition in overweight and obese populations. Our findings confirm that active exercise significantly improves both subjective sleep quality and BMI, though through distinct dose–response mechanisms: while combined aerobic and resistance training (ART, SUCRA ≈ 77.1%) and Resistance Training (RT, SUCRA ≈ 75.2%) were most effective for sleep enhancement, aerobic exercise (AE, SUCRA ≈ 74.0%) was the optimal strategy for BMI reduction. Notably, univariate meta-regression revealed no statistically significant linear relationship between PSQI improvement and either the magnitude of BMI reduction (coefficient = 0.078, *p* = 0.855) or total exercise dose (coefficient = −0.004, *p* = 0.803), suggesting that sleep benefits may stem more from the specific exercise modality rather than mere duration accumulation. In contrast, BMI improvement exhibited a significant non-linear (U-shaped) dose–response relationship, identifying a cumulative duration of 60–70 h as the optimal window beyond which diminishing marginal returns occur. Consequently, we recommend a stratified management strategy for personalized exercise prescriptions: for patients prioritizing sleep improvement, ART or RT should be prioritized without the need for excessive duration, whereas for those focused on weight loss, AE remains the superior choice with total volume controlled within the identified optimal window to efficiently break the “obesity–sleep disorder” vicious cycle.
