# Correction: Network meta-analysis and dose–response analysis of exercise on sleep quality and BMI in obese populations

**DOI:** 10.3389/fpubh.2026.1810853

**Published:** 2026-02-17

**Authors:** Frontiers Production Office

**Affiliations:** Frontiers Media SA, Lausanne, Switzerland

**Keywords:** body mass index, dose–response relationship, exercise, network meta-analysis, obesity, randomized controlled trials, sleep quality

The incorrect versions of Figures 8–10 were published. The correct versions appear below.

**Figure 8:**
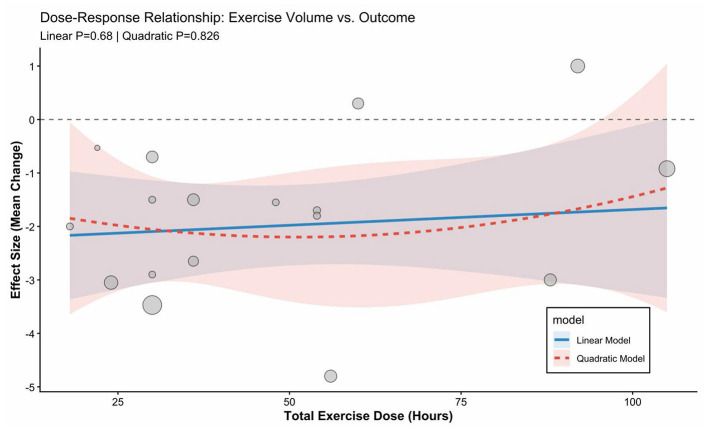
Meta-regression of total exercise dose and improvement in sleep quality.

**Figure 9:**
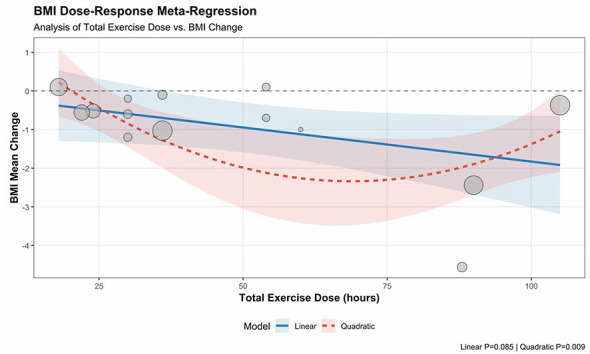
Meta-regression of total exercise dose and improvement in BMI.

**Figure 10:**
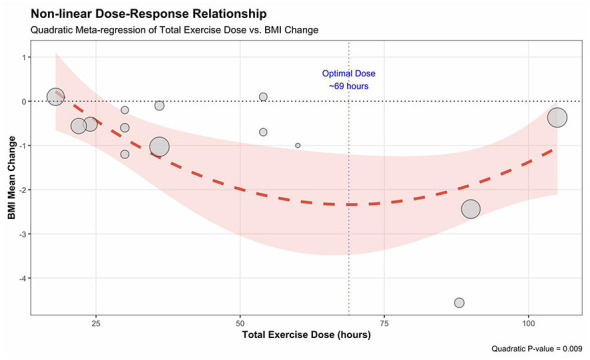
Non-linear dose–response relationship between total exercise dose and improvement in BMI.

The original version of this article has been updated.

